# Case report: Recurrent hemobilia due to hepatic artery pseudoaneurysm mimicking gastrointestinal tract bleeding

**DOI:** 10.3389/fmed.2022.1048763

**Published:** 2023-01-09

**Authors:** Jiaoyang Wang, Lei Yang, Lijun Xu, Lijie Qin, Yanwei Cheng

**Affiliations:** Department of Emergency, Henan Provincial People’s Hospital, People’s Hospital of Zhengzhou University, People’s Hospital of Henan University, Zhengzhou, China

**Keywords:** hemobilia, upper gastrointestinal bleeding, hepatic artery pseudoaneurysm, selective hepatic artery arteriography, case report

## Abstract

Hemobilia, a rare form of upper gastrointestinal bleeding (UGIB), is a potentially fatal complication that usually occurs after iatrogenic hepatobiliary trauma. However, hemobilia is clinically challenging to diagnose and often gets too late to diagnose. We herein report a case of recurrent hemobilia due to hepatic artery pseudoaneurysm (HAP) that was initially misdiagnosed as gastrointestinal tract bleeding. However, the patient was treated successfully with percutaneous coil occlusion of the pseudoaneurysm. This case illustrates that hemobilia can present as a mimic of gastrointestinal tract bleeding, but this is often difficult to diagnose at first glance and often misleads clinicians, especially emergency physicians, into making an incorrect diagnosis. Familiarity with the clinical features of hemobilia can help raise clinical suspicion and facilitate the early diagnosis and treatment of hemobilia.

## Introduction

Hemobilia is an uncommon but potentially life-threatening form of upper gastrointestinal bleeding (UGIB). It is defined as bleeding into the biliary system from a fistula between the splanchnic vasculature and any biliary duct, secondary to a variety of settings including accidental trauma, iatrogenic hepatobiliary injury, inflammation, and malignancy (1, 2). The clinical signs of hemobilia vary, with only 13 to 22% of patients presenting with the typical Quincke triad of symptoms consisting of right upper abdominal pain, jaundice, and UGIB (3, 4). Of note, UGIB is the most common sign of hemobilia and is present in 90% of patients; however, hematemesis and melena are usually mild and can stop spontaneously in most cases of hemobilia, which often cause patients to delay seeking medical attention until the bleeding is life threatening and must require emergency interventions (5, 6). In such an emergency case, it can often be very difficult to make a timely and correct diagnosis for clinicians, especially for emergency physicians, which may lead to delayed treatment.

Herein, we report the case of a 15-year-old boy with recurrent hematemesis and melena culminating in massive hematemesis and shock suggestive of gastrointestinal tract bleeding that was ultimately diagnosed with hemobilia due to hepatic artery pseudoaneurysm (HAP) rupture.

## Case presentation

A 15-year-old boy was admitted to our emergency intensive care unit (EICU) with the complaint of intermittent upper abdominal pain accompanied by hematemesis and melena for 1 month, which aggravated for 2 days. At the local hospital, the boy was initially misdiagnosed as an acute gastric mucosal lesion due to a history of taking non-steroidal anti-inflammatory drugs (NSAIDs) for a cold. However, the aforementioned symptoms then recurred every other week and were mild and can stop soon after receiving conservative treatment each time. Notably, 2 months before his admission to EICU, the boy accidentally crashed into an iron railing while riding his bicycle and then immediately developed right upper abdominal pain without hematemesis. He was admitted to another local hospital and underwent an abdominal computed tomography (CT) examination, which reported a high-density area with a diameter of approximately 6.5 cm in the right lobe of the liver. The boy was highly suspected of hepatic laceration. Finally, lacerations were observed in the posterosuperior segments (S7 and S8) of the liver and were repaired laparoscopically with sutures. Sadly, he failed to follow-up and return for checkup regularly. The patient had no relevant personal or family history.

Physical examination on admission showed abnormal vital signs with a body temperature of 37.7°C, a respiratory rate of 27 beats per minute (bpm), a heart rate (HR) of 135 bpm, a blood pressure (BP) of 105/55 mmHg, and a pale skin mucous membrane without jaundice and mild upper abdominal tenderness without mass. The results of laboratory tests showed reduced red blood cell count (RBC) of 1.85 × 10^12^/L (normal 4.3–5.8 × 10^12^/L) with 13.7% hematocrit (HCT) (normal 40–50%), reduced hemoglobin (Hb) concentration of 43.0 g/dl (normal 130–170 g/dl), and abnormal liver function with an albumin level of 30.0 g/L (normal 40–55 g/L), aspartate aminotransferase (AST) of 122.0 U/L (normal 15–40 U/L), alanine aminotransferase (ALT) of 179.0 U/L (normal 9–50 U/L), alkaline phosphatase of 182.0 U/L (normal 45–125 U/L), and glutamyltransferase of 252.0 U/L (normal 10–60 U/L). Total bilirubin, coagulation, and other laboratory results were within normal limits.

**FIGURE 1:**
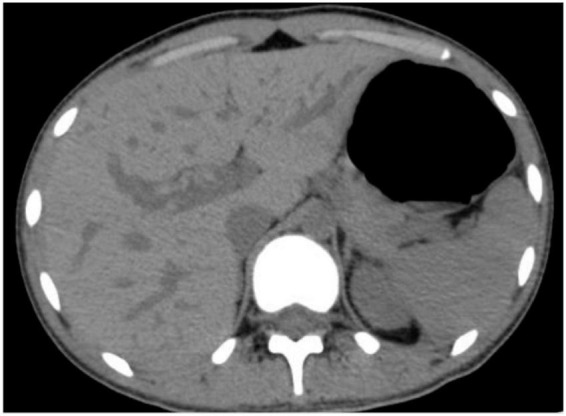
Preoperative abdominal computed tomography showing abnormal density shadows in the right hepatic lobe with possible hemorrhage.

**FIGURE 2:**
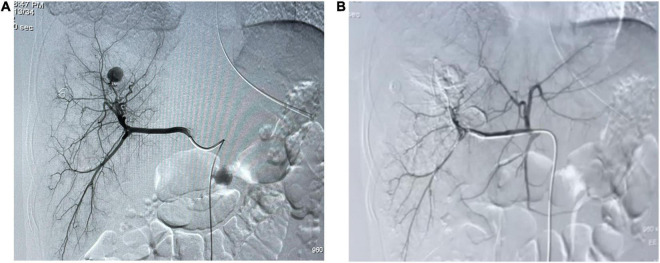
Arteriography. **(A)** Selective arteriography of the hepatic artery showing a sac-like pseudoaneurysm. **(B)** Angiographic embolization of the right hepatic artery showing the pseudoaneurysm disappeared.

**FIGURE 3:**
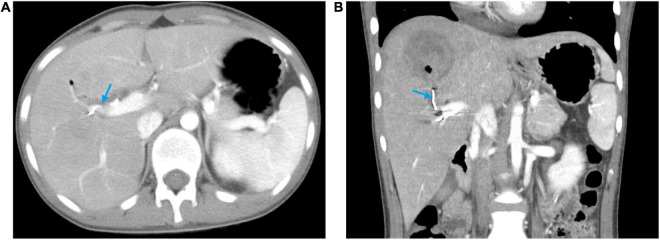
Abdominal contrast-enhanced computed tomography at discharge showing the pseudoaneurysm disappeared and the hyper-density coil (blue arrow). **(A)** Horizontal plane. **(B)** Coronal plane.

On the day of admission, he was treated conservatively with intravenous fluids, hemostatic and acid-suppressing drugs, and whole blood transfusion. However, subsequent to his admission, he developed massive hematemesis and melena, and his BP level decreased to 57/32 mmHg. In addition, repeated examinations of blood routine and coagulation revealed a severe abnormality. The patient was promptly given 4 units of RBC and 400 ml of plasma transfusion. An emergency esophagogastroscopy was performed, and bleeding was noted in the ampulla of Vater (Supplementary Video 1). Immediately thereafter, we performed an abdominal CT, which revealed abnormal density shadows in the right hepatic lobe with possible hemorrhage (Figure 1). All these findings together with the history of laparoscopic hepatic rupture repair prompted the suspicion of HAP rupture, which was further confirmed by the presence of a sac-like pseudoaneurysm beginning from the front branch of the right hepatic artery on selective hepatic artery arteriography (Figure 2A). Percutaneous coil occlusion of the right hepatic artery was performed through the ipsilateral femoral artery, and three coils (COOK MWCE-35-14-6-NESTER, MWCE-18-14-8-NESTER, and MWCE18-14-10-NESTER) were placed. Repeat arteriography showed that the pseudoaneurysm disappeared (Figure 2B). After the surgery, the hematemesis stopped, and his Hb level together with liver function turned normal gradually. An abdominal contrast-enhanced CT (CECT) showed no enhanced pseudoaneurysm when he was discharged (Figure 3). At the outpatient follow-up 2 months later, the patient became completely asymptomatic without hematemesis, melena, or jaundice, and his liver function was normal.

## Discussion

Hemobilia refers to the extravasation of blood into the biliary tract. While hemobilia represents only a small minority of UGIB and the bleeding is usually minor, it can be potentially lethal if not promptly recognized and treated. In the past, the most common cause of hemobilia was accidental trauma, accounting for 38.6% of all cases (7). Due to the expansion of interventional hepatopancreatobiliary procedures, iatrogenic causes have currently superseded other causes and have also contributed to an increased incidence of hemobilia. Green et al. reported that 144 out of 222 patients with hemobilia had an iatrogenic cause and only 13 patients had a traumatic cause (3). Classically, hemobilia presents with a combination of right upper abdominal pain, jaundice, and UGIB. However, most patients do not have the Quincke triad of symptoms but instead present with hematemesis and melena, which are typical manifestations of gastrointestinal tract bleeding. This can inevitably make it difficult for clinicians, especially emergency physicians, to differentiate between hemobilia and gastrointestinal tract bleeding, and even lead clinicians to make an incorrect diagnosis. Thus, hemobilia is clinically challenging to diagnose and is often diagnosed too late.

Hereby, the case presented a boy with recurrent hemobilia who was initially misdiagnosed with acute gastric mucosal lesion due to a history of taking NSAIDs 1 day prior to onset. However, taking NSAIDs for 1 day is unlikely to cause bleeding, and the local doctor lost sight of the history of laparoscopic liver repair and the suspicion of hemobilia. The boy presented with recurrent acute hematemesis and melena. Notably, the symptoms appeared to be periodic, occurring every other week. A previous study concluded that 85% of patients with hemobilia will experience periodic bleeding due to repeat blood coagulation and dissolution (1). It is a remarkable fact that the boy did not develop jaundice, which may be related to the rate of bleeding. Slow bleeding has a tendency to form clots in the bile duct and cause biliary obstruction because the blood does not mix with the bile and forms separate layers due to the difference in gravity and surface tension (8). Conversely, in the case of rapid bleeding, the blood flows rapidly into the duodenum and manifests as hematemesis and melena. Hemobilia is usually mild and can stop spontaneously in most cases, but it can at times be massive and present as an acute life-threatening condition. As in our case, the boy initially experienced recurrent minor bleeding but subsequently developed massive bleeding that resulted in shock. Fortunately, the history of laparoscopic hepatic rupture repair provided us with a hint of HAP rupture, which was also confirmed by selective hepatic artery arteriography showing a sac-like pseudoaneurysm.

Pseudoaneurysm develops from damage to an arterial wall, resulting in the rupture and formation of a sac walled by surrounding tissues and organs (9, 10). HAP is mostly caused by direct vascular injury due to accidental or iatrogenic hepatobiliary trauma (11). Due to changes in the current medical environment, iatrogenic injury has replaced hepatic trauma as the main cause of pseudoaneurysm of the hepatic artery (12, 13). Among all HAPs, the right hepatic artery is the most common site, accounting for almost 80% of all cases (12). On our selective hepatic artery arteriography, pseudoaneurysm starts from the front branch of the right hepatic artery. A rupture of HAP is a very rare cause of hemobilia and occurs usually 4 weeks following the iatrogenic injury (14, 15). In the present case, the boy also presented with hemobilia 1 month after undergoing a laparoscopic hepatic rupture repair. The rupture of HAP is considered an acute emergency with an associated mortality of up to 50% and requires immediate intervention (16). An urgent selective hepatic arterial arteriography and embolization are now recommended as the first line of treatment for controlling arterial bleeding in hemobilia due to its success rate of over 80%, good tolerance, and minimal risk (3, 17). Compared with surgical intervention, embolization showed 25% lower mortality and 67% less morbidity (18). However, surgery should be considered if embolization fails, pseudoaneurysms are infected, or other vascular structures are compressed (2). In addition, vascular stenting seems to be a promising alternative to embolization, which can maintain luminal patency and thus avoid liver necrosis complication (19). However, most stents could not be implanted satisfactorily because of the tortuous access of the right hepatic artery and the poor compliance of the stent. Consistent with the previous similar case reported by Bains (20), we also performed emergency arterial embolization. Postoperatively, the boy was completely asymptomatic and there was no enhancing pseudoaneurysm on abdominal CECT.

## Conclusion

Hemobilia is a potentially life-threatening form of UGIB. Hemobilia must be suspected in any patient with unclear UGIB, especially if accompanied by periodic bleeding or jaundice, and with a recent history of hepatobiliary intervention or hepatic trauma. Selective hepatic arterial arteriography and embolization remains the mainstay diagnostic and therapeutic option in significant hemobilia.

## Data availability statement

The original contributions presented in this study are included in the article/Supplementary material, further inquiries can be directed to the corresponding authors.

## Ethics statement

The studies involving human participants were reviewed and approved by Ethics Committee of Henan Provincial People’s Hospital. Written informed consent to participate in this study was provided by the participants’ legal guardian/next of kin. Written informed consent was obtained from the individual(s), and minor(s)’ legal guardian/next of kin, for the publication of any potentially identifiable images or data included in this article.

## Author contributions

JW and LY were the patient’s physicians and collected the data for case presentation. YC conducted the literature search and prepared the first draft of the manuscript. LQ contributed to the final manuscript drafting. LX reviewed the manuscript and suggested editing. All authors contributed to the article and approved the submitted version.

## Supplementary material

Supplementary Video 1 Esophagogastroscopy that shows bleeding in the ampulla of Vater.
